# The Enigmatic Nature of the TCR-pMHC Interaction: Implications for CAR-T and TCR-T Engineering

**DOI:** 10.3390/ijms232314728

**Published:** 2022-11-25

**Authors:** D. V. Shevyrev, V. P. Tereshchenko, S. V. Sennikov

**Affiliations:** 1 Laboratory of molecular Immunology, Research Institute for Fundamental and Clinical Immunology, 630099 Novosibirsk, Russia; 2 Center for Cell Technology and Immunology, Sirius University of Science and Technology, 354340 Sochi, Russia

**Keywords:** TCR, mechanosensor, pMHC, affinity, CAR-T, TCR-engineering, Ag-recognition, kinetic proofreading, optical tweezers, TCR mechanobiology, tangential force, shear stress, FG-loop, energy

## Abstract

The interaction of the T-cell receptor (TCR) with a peptide in the major histocompatibility complex (pMHC) plays a central role in the adaptive immunity of higher chordates. Due to the high specificity and sensitivity of this process, the immune system quickly recognizes and efficiently responds to the appearance of foreign and altered self-antigens. This is important for ensuring anti-infectious and antitumor immunity, in addition to maintaining self-tolerance. The most common parameter used for assessing the specificity of TCR-pMHC interaction is affinity. This thermodynamic characteristic is widely used not only in various theoretical aspects, but also in practice, for example, in the engineering of various T-cell products with a chimeric (CAR-T) or artificial (TCR-engineered T-cell) antigen receptor. However, increasing data reveal the fact that, in addition to the thermodynamic component, the specificity of antigen recognition is based on the kinetics and mechanics of the process, having even greater influence on the selectivity of the process and T lymphocyte activation than affinity. Therefore, the kinetic and mechanical aspects of antigen recognition should be taken into account when designing artificial antigen receptors, especially those that recognize antigens in the MHC complex. This review describes the current understanding of the nature of the TCR-pMHC interaction, in addition to the thermodynamic, kinetic, and mechanical principles underlying the specificity and high sensitivity of this interaction.

## 1. Introduction

The adaptive immunity that emerged during evolution endows the immune system with a number of advantages [1,2]. The sensitivity and specificity of antigen recognition, in addition to the possibility of immunological memory formation, are, apparently, considered to be the most significant of them [3,4]. The specificity or ability of immunity to distinguish and recognize antigens with high accuracy [5] is associated with the emergence of mechanisms for diversifying the repertoires of antigen-recognizing receptors [6,7], with the emergence of selection mechanisms [8,9,10,11], and with the formation of the major histocompatibility complex (MHC) [12,13,14]. Thus, due to the genome rearrangement, a colossal set of antigen-recognizing receptors sufficient to cover the potential diversity of antigens is being preventively formed [15,16,17,18,19,20,21,22]. At the same time, the stages of positive and negative selection provide the ability to distinguish between self- and non-self-antigens, while the MHC system, in addition to the presentation of foreign antigens, allows distinguishing between self- and altered self-antigens [3,23,24]. The latter, apparently, demonstrate a significant evolutionary advantage, allowing the immune system to more efficiently execute antitumor surveillance and fight various infections [3,25,26,27,28,29,30].

The emergence of such mechanisms requires significant genetic transformations. It is assumed that two macroevolutionary events resulted in the emergence of modern adaptive immunity in vertebrates [2,31,32]. The first of these events was the “molecular domestication” of the ancient transposon of recombination-activating gene (*RAG*), which occurred approximately 890 million years ago and led to the emergence of *RAG1*, *RAG2*, and recombination signal sequences (*RSSs*) in jawed vertebrates, and *RAG1L* and *RAG2L* genes in invertebrates [32,33]. The second evolutionary leap was associated with two genome-wide duplications that occurred sequentially approximately 650–550 million years ago [31,34]. These events determined the further evolutionary development of the anatomical structures and physiology of adaptive immunity [1,2,35,36]. Thus, at the stage of jawed vertebrates, the thymus, spleen, and lymph nodes (in warm-blooded animals) emerged, the system of processing and presentation of antigens developed, and the mechanisms of selection (AIRE expression in thymus) and diversification of the repertoires of antigen-recognizing receptors were improved [1,2,37]. During phylogenesis, the co-evolution of the T-cell receptor (TCR) and MHC genes also occurred, which provided a finer “calibration” and an increase in the accuracy of the interaction between TCRs and MHC molecules in jawed chordates [38,39,40]. Thus, more than half a billion years of evolution, large-scale genetic transformations, and significant anatomical and physiological changes lie behind the precision specificity of the antigen recognition that is characteristic of the immunity of higher chordates.

At present, the molecular mechanisms of the synthesis of MHC molecules and TCRs are relatively well studied, their structure has been established, and 3D structures of various TCR-pMHC complexes have been obtained [14,41,42,43,44,45,46]. Billions of unique amino acid sequences of TCRs have been deciphered due to high-throughput sequencing technology, and modern data analysis methods have provided numerous opportunities for studying various properties of TCR repertoires [7,16,17,18,19,20,21,22]. However, despite intensive research in this area, the nature of the paradoxical specificity of the TCR-pMHC interaction remains largely unclear. This review reveals modern ideas about the nature of high specificity and sensitivity, based on the thermodynamic, kinetic, and mechanical principles of the TCR-pMHC interaction.

## 2. Thermodynamic Aspect

In order to reveal the specificity of a particular TCR-pMHC interaction pair, the concept of affinity is used [47,48,49], implying that the complementarity between TCR and pMHC underlies the discrimination of the correct and wrong target [50]. Affinity is a thermodynamic expression of the binding strength of molecules interacting based on stereochemical correspondence, which can be expressed in terms of the binding constant ($K_{a}$):

(1) $$\mathrm{TCR}+\mathrm{pMHC}\begin{array}{c} k_{\mathrm{on}}\\ ⇄\\ k_{\mathrm{off}} \end{array}\mathrm{TCR}\text{-}\mathrm{pMHC}\;$$

(2) $$K_{a}=\frac{\left[\mathrm{TCR}\text{-}\mathrm{pMHC}\right]}{\left[\mathrm{TCR}\right]\times \left[\mathrm{pMHC}\right]}=\frac{k_{\mathrm{on}}}{k_{\mathrm{off}}}$$

where $k_{\mathrm{on}}\;\mathrm{and}\;k_{\mathrm{off}}$ are the rate constants of association and dissociation reactions, respectively [51,52,53]. In this representation, the free energy (ΔG) of the bond should determine the specificity of the TCR-pMHC interaction:

(3) $$\Delta G=-{\mathrm{RTlnK}}_{a}$$

where R is the universal gas constant and T is the absolute temperature [54,55,56]. The specificity of the interaction in this case is provided by thermodynamic selectivity ($S_{\mathrm{td}})$, which can be expressed in terms of the ratio of the binding constants of competing ligands:

(4) $$S_{\mathrm{td}}=\frac{K_{a\;\left(\mathrm{pMHC}\;1\right)}}{K_{a\;\left(\mathrm{pMHC}\;2\right)}}=\frac{e^{-\frac{{\Delta G}_{\left(\mathrm{pMHC}\;1\right)}}{\mathrm{RT}}}}{e^{-\frac{{\Delta G}_{\left(\mathrm{pMHC}\;2\right)}}{\mathrm{RT}}}}$$

where pMHC 1 and pMHC 2 are the competing ligands, and *e* is the base of the natural logarithm [57].

However, the difference in free energy between the correct and wrong target may be insignificant or absent [58], which significantly limits the use of affinity for determining the specificity of the TCR-pMHC interaction [59,60]. A similar paradox is well known in biochemistry for many molecular interactions; for example, during protein synthesis, each mRNA codon selectively interacts with a specific tRNA anticodon [59,61]. In this case, the error rate is ~1 in 20,000; this is unattainable only due to the difference in affinity because, between anticodons that differ by one base, the difference in free energy is too small to provide a similar level of accuracy [62,63]. This contradiction was resolved in the mid-1970s, when John Hopfield and Jacques Ninio almost simultaneously proposed the kinetic proofreading model [60,64]. This model took into account the multistage nature of ligand–receptor interactions, when there were many irreversible steps, each of which consumed energy and increased specificity [65,66,67]. This model included the kinetics of ligand–receptor interactions and provided sufficient explanations for error correction during protein synthesis, amino acid binding to tRNA, and DNA replication [68]. Subsequently, the kinetic proofreading model was supplemented and adapted to describe the TCR-pMHC interaction.

## 3. Kinetic Aspect

**Figure 1:**
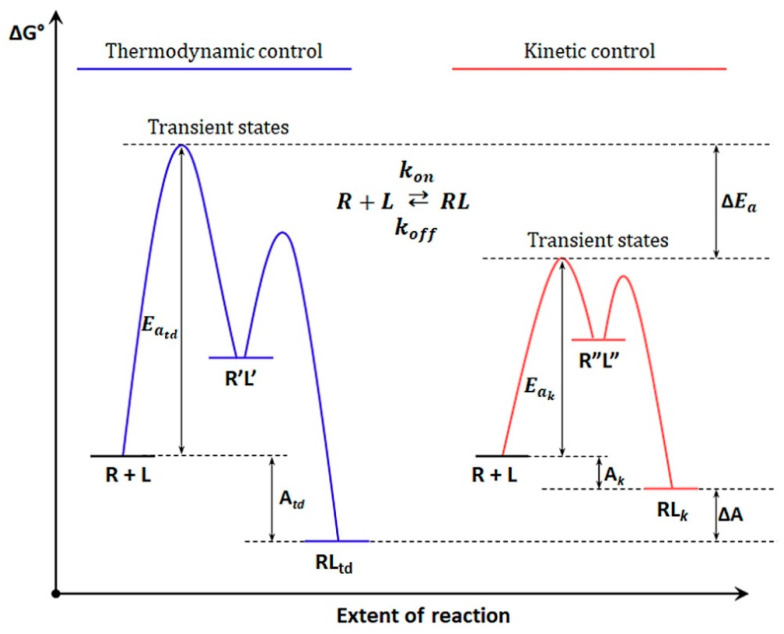
An example of an energy diagram. The difference in relative free energies between stable and transient states under conditions of thermodynamic and kinetic control is demonstrated. ΔG° is the Gibbs energy; R is the receptor (TCR); L is the ligand (pMHC); $E_{a_{td}}$ and $E_{a_{k}}$ represent the activation energies or energy barriers that the system overcomes due to the energy of thermal fluctuations in the case of thermodynamic and kinetic controls, respectively; A is the affinity; ΔA is the difference in affinity; $\Delta E_{a}$ indicates, in this example, a higher probability of the thermodynamic state of the system under kinetic control; _k_ is the kinetic control; _td_ is the thermodynamic control; R’L’ and R”L” indicate different reaction intermediates.

In biological systems, the kinetics of ligand–receptor interactions is no less significant than affinity for the reason that different processes require time [58]. Equation (2) shows that the affinity expressed in terms of the association constant is equal to the ratio of the two rate constants. Therefore, with the same affinity, the reaction kinetics can be different if the association and dissociation rates are proportionally different [56,58]. The $k_{\mathrm{on}}\;\mathrm{and}\;k_{\mathrm{off}}$ rates are determined by the difference in free energy between stable and transient states, whereas affinity is the difference in free energy between unbound and bound state (Figure 1). As noted above, various processes, whether enzymatic reactions or TCR activation, require time, i.e., the interaction efficiency depends on the stability of the ligand–receptor complex [58,69]. Quantitatively, stability is expressed by the complex lifetime or residence time, which is inversely proportional to the dissociation constant:

(5) $$\tau =\frac{1}{k_{\mathrm{off}}}.$$

The residence time depends on a combination of various factors that affect the relative free energies of the stable and transient states, and it follows from Equations (2) and (5) that low affinity is not always associated with short residence time [56,58,70]. Thus, along with affinity, the specificity of the interaction in many irreversible processes is provided by kinetic selectivity ($S_{k}$):

(6) $$S_{k}=\frac{k_{\mathrm{on}\;\left(\mathrm{pMHC}\;1\right)}}{k_{\mathrm{on}\;\left(\mathrm{pMHC}\;2\right)}}\;$$

where pMHC 1 and pMHC 2 are the competing ligands [58].

**Figure 2:**
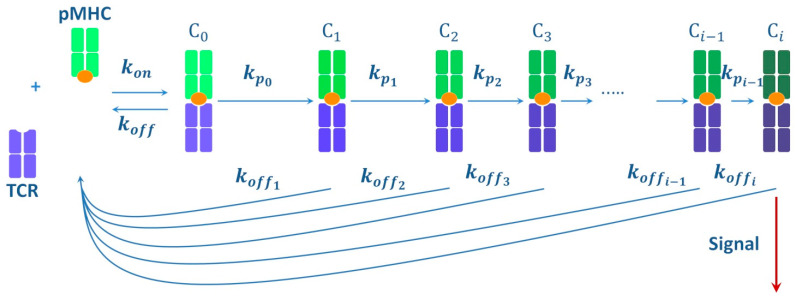
Diagram of one of the kinetic proofreading models. The TCR and pMHC molecules bind forming the initial C_0_ complex; this process corresponds to Equation (1) with the $k_{\mathrm{on}}{\;\mathrm{and}\;k}_{\mathrm{off}}$ rate constants. The formation of the C_0_ complex triggers kinetic proofreading with successive stabilization of the complexes from C_0_ to C_i_ and an increase in the propagation rate from $k_{p_{0}}$ to $k_{p_{i}}$. This happens when the correct target is recognized. If the target is wrong, there is a rapid dissociation of the complex to its original state, when TCR and pMHC are not associated.

The kinetic understanding of specificity established the basis of kinetic proofreading models, which were further adapted for describing the TCR-pMHC interaction [71]. Figure 1 demonstrates the integral energy effects of direct and reverse reactions. In reality, the energy profiles of reactions are much more complex and consist of many small energy barriers that the system overcomes due to the energy of thermal vibrations, passing through a number of metastable states [72]. The process is followed by conformational changes in the ligand–receptor system, in addition to various thermodynamic effects. This is also applied to the TCR-pMHC interaction [73]. In the case when the transition to each subsequent conformational state of the TCR-pMHC complex is irreversible, a proofreading chain is formed, which comprises successive transitions in a series of intermediate conformational states of the TCR-pMHC complex (Figure 2) [66,71,74,75]. In such a case, there is no return to the previous conformational state. Only the dissociation of the complex to the initial unbound state of the receptor (TCR) and the ligand (pMHC) is possible, which provides the proofreading process, when wrong targets do not achieve the necessary stereochemical correspondence with the receptor and leave the proofreading chain without causing TCR activation [66,71,75]. Correct targets move further along the chain of transient states until the formation of the TCR-pMHC signaling complex, ensuring signal transmission into the cell [66,71,74,75]. It is assumed that such a multistage nature allows the enhancement of the difference between correct and wrong targets due to the energy advantage, which manifests itself in the summation of thermodynamic effects occurring upon transition to each subsequent conformational state [58,60,72,74]. This also allows increasing the time during which the receptor is in the bound state with the correct target, which induces the transmission of the activation signal [58,74,76,77,78]. Therefore, high specificity can be achieved by a sufficiently large number of steps in the proofreading chain.

The TCR-pMHC interaction is central to the adaptive immune response. Therefore, in addition to specificity, it should have high sensitivity so that T cells can detect sparse foreign antigens among many self-antigens [79]. However, the calculations provided when testing the kinetic proofreading model revealed the presence of a trade-off between specificity and sensitivity, i.e., an improvement in specificity due to an increase in the number of steps in the proofreading chain occurred at the expense of a decrease in sensitivity, and vice versa [71,80]. Attempts to resolve this contradiction resulted in the emergence of several models. They included additional parameters, mainly considering any changes in the stability of the TCR-pMHC complex in the activation chain or various conditions for controlling signal transmission from the TCR [59,67,81,82,83,84,85,86]. For example, one of the models, which included sequential stabilization of the complex and chain propagation in the case of a correct target, and vice versa in the case of a wrong target, showed a dramatic increase in both specificity and sensitivity (Figure 2) [75]. This model also demonstrated that it took more than 6 s to implement kinetic proofreading, and starting from 10 s, there was no significant increase in the sensitivity and specificity of recognition [75]. The prediction was found to be quite accurate—optogenetic studies on living cells confirmed that the optimal time for the kinetic proofreading implementation was 8 s [76,87]. It should be noted that a comparative analysis of most of these models revealed a common pattern: T lymphocyte activation depended more on individual values of $k_{\mathrm{on}}{\;\mathrm{and}\;k}_{\mathrm{off}}$ than on the $k_{\mathrm{on}}/k_{\mathrm{off}}$ ratio, i.e., reaction kinetics contributed more to specificity than thermodynamics [59].

Therefore, various theoretical models and experiments on living cells have demonstrated that the kinetic proofreading and stability of the TCR-pMHC complex determine the specificity of the interaction and affect the signal transmission more than the affinity.

## 4. Mechanical Aspect

**Figure 3:**
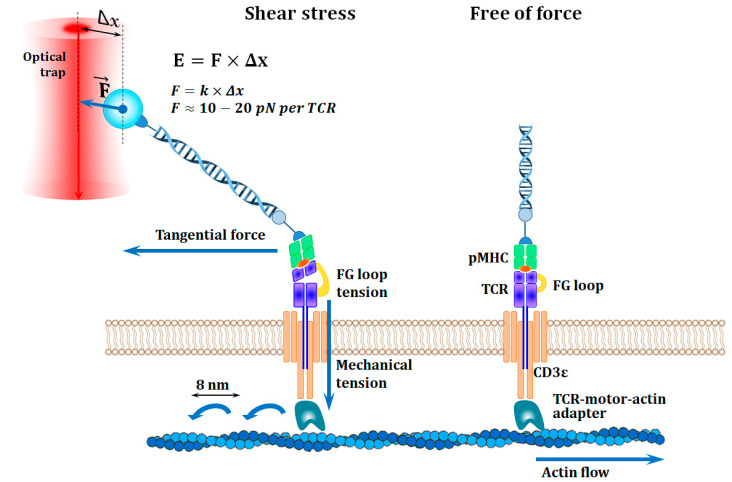
The diagram demonstrates the action of two forces causing the activation of the T-cell mechanosensor. Movement of the T lymphocyte along the APC after the binding of TCRs to pMHC molecules results in a tangential force. Quaternary structure of the TCRαβ heterodimer changes due to the FG-loop stretching, through which mechanical energy is transferred into the cell by means of the CD3εγ and CD3εδ chains. Internal strength arises from the movement of actin filaments and the rearrangement of the cytoskeleton. The adapter connection of CD3 chains with actin filaments is movable, and the pitch is approximately 8 nm, which allows compensating for the excess tension in the system and ensures the lifetime of the TCR-pMHC complex necessary for the activation. The shear force required for activating a T lymphocyte with a single TCR molecule is ~10–20 pN.

The proposed proofreading models have allowed good predictions of the residence time and explained various energetic aspects of the specificity of the TCR-pMHC interaction. However, the mechanisms of specific TCR activation varied between models, which did not allow even a verbal formulation of a unified hypothesis of antigen recognition by T cells [60]. The situation was improved when it was suggested that the kinetics of antigen recognition and signal transmission from the TCR were regulated by mechanical forces, and the TCR itself was a mechanosensor (Figure 3) [88]. A pioneering study in 2009 clearly demonstrated that the TCR, when being activated, converted mechanical energy into biochemical signals [88]. This occurred when a T lymphocyte interacted with an antigen-presenting cell (APC) and the TCR bound to the cognate pMHC complex [88,89]. In this case, tangential force began to act on the TCR due to the displacement of cells relative to each other [89]. Shear stress caused a change in the quaternary structure of the variable part of the TCRαβ heterodimer, which transmitted torque to the rigid system of CD3εγ and CD3εδ chains [89,90]. Thus, mechanical energy was spent on changing the spatial position of intracellular domains, which resulted in cascade of biochemical reactions and induced activation of the T cell [88,89,90,91].

During antigen screening, the T cell polarizes with lamellipodia and microvilli being formed at the leading pole, and the cell continues to move [92]. At this time, TCRs bound at the leading edge move to the rear part, while experiencing the action of an internal force caused by actin retrograde flow [93,94]. This leads to a slowdown in cell movement, and the bound TCRs are collected at the posterior pole, where the formation of the immune synapse begins [90,95]. Considering the dynamics of the process, it is sometimes called an immunological kinapse [95]. The external energy of the shear stress and the energy of the actin retrograde flow act in total on the mechanosensory complex [90,96]. These forces create a total force estimated at 10–20 pN/TCR, corresponding to the threshold of T lymphocyte activation [90,97]. It should be noted that the TCR-motor–actin system can compensate for excess stress in the mechanosensor complex, maintaining a balance in the threshold range [90,98]. This prevents premature breakdown of the TCR-pMHC complex, in addition to providing time for structural and conformational changes necessary for TCR activation [90,96].

The processes described above do not provide an intuitive understanding of the increase in sensitivity and specificity of antigen recognition by the TCR. High sensitivity in this context implies the ability of T cells to discern sparse foreign antigens among the many self-antigens on the cell surface [90]. While considering the recognition process more thoroughly, it can be hypothesized that a smaller number of cognate pMHCs on the APC surface focuses shear stress and increases the force acting on a particular TCR-pMHC complex [90,99,100]. Furthermore, the larger number of self-pMHCs on the APC surface leads to shear stress distribution among many TCR-pMHC complexes, reducing the force acting on each TCR individually [90,99,100]. This understanding of mechanical enhancement of sensitivity has been confirmed in elegant experiments using optical tweezers and is consistent with a number of observations supporting the possibility of T lymphocyte activation by a signal from only one TCR [90,99,100].

The external energy used in antigen recognition implies that the TCR-pMHC interaction is a nonequilibrium process [90,101]. In the equilibrium system of Equation (1), free transitions between the bound and unbound states are provided by the energy of thermal fluctuations ($K_{B}$T = 4.1 pN·nm). In a nonequilibrium process, a larger energy barrier needs to be overcome due to the supply of energy from outside [90,101]. In this case, the reaction rate is determined by the Arrhenius equation and depends exponentially on the amount of energy ($E_{a}$) that is supplied to the system:

(7) $$k=A\times e^{-\frac{E_{a}}{K_{B}T}}$$

where k is the reaction rate, A—the frequency factor, e—the base of the natural logarithm, $E_{a}$—the activation energy, $K_{B}$—the Boltzmann constant, and T—the absolute temperature. Using optical tweezers, it was found that the TCR activation energy was ~150 pN·nm, which was about 37 times greater than the energy of thermal fluctuations. This allows colossal energy gain of $e^{37}$. Some of the energy will be lost. However, if at least one-quarter of this energy is used by the mechanosensor, then the gain will be four orders of magnitude greater than in the equilibrium process [55,90]. Given that affinity is determined in an equilibrium process, this imposes additional restrictions on the use of this parameter for explaining the sensitivity of the TCR-pMHC interaction. Thus, the mechanical nature of TCR allows detecting sparse and even single epitopes in MHC molecules due to a large energy advantage. This gives the immune system an exceptionally high sensitivity to detect various threats.

A number of interesting experiments underlie the concept of the mechanical nature of the specificity of antigen recognition by the TCR. Thus, a paradoxical increase in the strength and lifetime of the TCR-pMHC bond under the influence of force to recognize the cognate antigen was found [90,102,103]. Furthermore, conversely, accelerated breakdown of the TCR-pMHC complex under the influence of force in the case of nonspecific interaction was established. Such bonds have been called catch bonds and slip bonds, respectively [90,102,103]. Under these conditions, allosteric mechanisms control the strength and duration of the bond, which is confirmed by the model where a change in the quaternary structure of the TCRαβ heterodimer regulates antigen recognition in the MHC complex under the influence of shear forces [100,102,103,104]. In this case, thermodynamics provides the primary interaction between TCR and pMHC, when the association and dissociation of the complex occur due to the energy of thermal fluctuations, and the ratio of the rates of these processes determines the affinity. At the same time, the application of an external tension force acting on the complex due to cell movement or actin traction will determine the specificity of the interaction, strengthening or weakening the bond strength depending on the change in the spatial configuration [94,98,100,102,103,104]. This means that slip bonds are initially formed for all TCR-pMHC interactions, which is determined by the thermodynamics of the process. However, the slip bond is only transformed into a capture bond for agonist ligands, and transfers force to the mechanosensor, which is increased with the increase in the applied external force [90,102,104]. With a further increase in the external force (>15 pN), the probability of bond breaking also increases. In the case of inappropriate ligands, the application of an external force breaks the slip bond much faster and no TCR activation occurs [90,102]. However, it should be noted that such sliding interactions enable the transmission of tonic signals that are necessary for the homeostatic maintenance of the pool of T lymphocytes [105,106,107].

The formation of catch bonds does not occur between individual TCR and pMHC molecules, but was observed only on living cells, where TCRs were mechanically coupled to actin and other elements of the cytoskeleton [90,108]. This emphasizes the special role of the cytoskeleton in the formation of the mechanosensory apparatus of T lymphocytes.

Therefore, the mechanical nature of TCR provides exceptional specificity and sensitivity for recognition of sparse antigens in the MHC complex, in addition to resistance of effector T lymphocytes to self-antigens.

## 5. Practical Value

In recent years, understanding of the mechanisms underlying the specificity of antigen recognition by T lymphocytes has significantly expanded. However, current ideas about the TCR mechanobiology are considered to be incomplete, establishing the need for further research of this issue in order to create more efficient cell therapy products [109].

The trade-off between specificity and sensitivity is now well known. However, at the dawn of the CAR-T technology development, significant efforts were directed to obtaining receptors with high affinity [110]. Later, it was found that the use of CARs with low micromolar affinity can provide excellent antitumor efficacy and high safety with a low risk of systemic non-tumor toxicity compared to high-affinity CARs [111,112,113,114]. This was explained by the low specificity of CAR-T cells with high nanomolar affinity, which resulted in their nonspecific activation and the occurrence of systemic toxicity or a complete lack of effect [113]. Such CAR-T cells with excessive affinity of the receptor can be depleted more quickly and lose their efficiency [115,116].

By analogy with natural lymphocytes, the CAR receptor must be mechanically coupled to the cytoskeleton (through CD3ζ) for successful antigen recognition and cytotoxic function [109]. Recent studies have shown that the CAR receptor is also a mechanosensor; the force required for its activation is higher than that of natural T cells and lies in the range of 60–100 pN [117]. However, the thermodynamic aspect of antigen recognition is more important for the CAR receptor than for the naturally occurring TCR. This is confirmed by the fact that CAR receptors only form slip bonds with cognate antigens, and the probability of their breaking increases with an increase in the applied external force [118].

When sparse intracellular antigens become the target, the use of artificial TCR-T lymphocytes is more preferable and has a number of advantages that result from the high specificity and sensitivity of natural TCRs [119]. At the same time, the use of artificial TCRs with high affinity for the target antigen, similar to CAR-T, can lead to a decrease in recognition efficiency, nonspecific activation, and early depletion of T cells [120,121]. It should be noted that the affinity of natural TCR receptors is significantly lower than that of antibodies and lies in the millimolar range [109,122]. However, this does not reduce the sensitivity and specificity of antigen recognition in the MHC complex due to the use of external mechanical forces. The ability to activate TCRs is reflected in the distance between TCR and pMHC molecules: for super-agonists, it is 44 ± 9 Å; for normal agonists, it is 54 ± 11 Å; and for weak agonists, it increases to 66 ± 18 Å [100].

In addition, it is known that the physicochemical properties of TCRs differ between different populations of T cells [123]. For example, the associations between the genetic variability of MHC-I and the expression profiles of V-genes of TCRs of CD8^+^ lymphocytes are more significant in comparison with similar associations for CD4^+^ cells [124]. This is explained not only by the processes of coevolution [40], but also by closer contact between the V regions of the β-chain of TCRs of CD8^+^ cells and complementary regions of MHC-I molecules, which is determined by the difference in the spatial organization of TCRs of CD4^+^ and CD8^+^ lymphocytes [125,126,127]. This difference is established during maturation in the thymus, when the choice between CD4 and CD8 is determined by the TCR affinity for the corresponding class of MHC molecules [128]. Studying characteristics of the CDR3 loop, such as hydrophobicity, loop length, predicted mean free binding energy of TCR-pMHC, and some other parameters, revealed differences in the physicochemical properties of TCRs at the level of different populations [123]. Thus, regulatory T cells bind their pMHC ligands less specifically and have a lower mean TCR-pMHC binding energy than T follicular helper cells, which demonstrate high specificity and have higher TCR-pMHC binding energy [123]. Similar differences are observed in other subpopulations. Thus, the physicochemical characteristics of the CDR3 loop among the Th1/Th1-17/Th17 populations were similar to those of T follicular helpers, whereas among the Th22/Th2a/Th2 populations there was a similarity with regulatory T cells [123]. Apparently, such differences impact not only the thermodynamics of antigen recognition, but also the mechanical features of this process. In the future, these differences will probably be considered when developing cell products aimed at replenishing the functions of different cell subpopulations. They may be used, for example, in the development of TCR-T therapy for autoimmune diseases based on the principles of regulatory T cells.

Thus, when developing new CAR-T or TCR-T products for cell therapy, the mechanical nature of antigen recognition should be considered, and parameters such as the force causing receptor activation, the distance between the receptor and the target, and the coupling of the receptor to the cytoskeleton (for example, by means of CD3ζ chain) should be evaluated. The thermodynamic and kinetic components should also be taken into account, not for maximizing affinity, but for assessing the potential feasibility of a ligand–receptor interaction and possibly determining the complex lifetime.

## 6. Conclusions

The development of sophisticated tools including various methods of microscopy (ultra-high resolution, atomic force, traction force, etc.), the emergence of optical tweezers, the adaptation of microfluidics systems, the use of 3D cultures, and the development of molecular tension probes and other methods have significantly changed the idea of the nature of antigen recognition by T cells [90]. Over the past 30 years, various hypotheses have emerged that explained the high sensitivity and specificity of the TCR-pMHC interaction [59]. The theories of sequential interaction, clustering and aggregation of TCRs, kinetic segregation, and kinetic proofreading were once widely accepted [59,90]. However, all of these required internal compromises or did not yield sufficient predictions of the energy or interaction time. The theory of kinetic proofreading is still considered to be suitable and provides a thorough understanding of the interaction kinetics [74,87,129]. However, the realization of the non-equilibrium process, and the need to use external energy to enhance the specificity and sensitivity of recognition, allow for a better understanding of the nature of this interaction, which is confirmed in advanced studies on living cells [76,90,130]. It is obvious that modern research devoted to the development of products with an artificial T-cell receptor should, along with the thermodynamic aspect, consider the kinetics and mechanics of the interaction of a synthetic receptor with an antigen, which will increase the specificity and efficiency of therapy.

In this review, we focused on the nature of the specificity of antigen recognition by the T-cell receptor. We described the thermodynamic, kinetic, and mechanical aspects of the interaction between TCR and pMHC, and their role in providing the specificity and sensitivity of this interaction. We also focused on resolving the paradox of both high specificity and sensitivity of this interaction, and the paradox of slip and catch bond formation. It should be noted that the productive interaction of TCR and pMHC lies at the early stage of T-lymphocyte activation. Then, the formation of an immune synapse begins with the engagement of many molecules, such as CD4, CD8, CD28, CD6, CD80/86, LFA-1, and phosphatase CD45 [131]. The central and peripheral microclusters are formed due to a spatial change in actomyosin cytoskeleton. All these changes lead to the recruitment of Lck kinases, and phosphorylation of ITAMs, to which ZAP70 binds and recruits LAT with the signalosome formation [131]. Thus, the activation amplitude, proliferation, exhaustion, or senesce of T cells are determined by the adequacy of stimulation and the conditions for the formation of an immune synapse [132,133]. Therefore, modern research devoted to the development of products with an artificial T-cell receptor should, along with the thermodynamic aspect, consider the kinetics and mechanics of the TCR-pMHC interaction, in addition to other molecules engaged in this interaction. This will increase the specificity and efficiency of therapy.

## Data Availability

Not Applicable.
